# High rate of hypoglycemia in diabetic pregnant women on use of glyburide

**DOI:** 10.1186/1758-5996-7-S1-A85

**Published:** 2015-11-11

**Authors:** Giordanna De Bacco, Vanessa Krebs Genro, Cristiano Caetano Salazer, Maria Lúcia R Opperman

**Affiliations:** 1 HCPA, Porto Alegre, Brazil

## Background

The prevalence of gestational diabetes (GD) in Brazil can exceed 18% when applied the current WHO diagnostic criteria. The importance of the GD treatment in reducing severe outcomes of perinatal maternal morbidity and has been shown in randomized clinical trials. Therapeutic options for the GD have included oral antidiabetic agents glibenclamide (GB) and metformin (MF) showed Results of safety and efficacy similar to insulin.

## Objective

To analyze the effects of the use of GB and MF in the treatment of GD in pregnant women at a specialized clinic in HCPA in a randomized open-label study.

## Materials and methods

Eighty-one women diagnosed with GD and drug treatment indication were randomized. The initial dose of GB was 2.5 mg/day with an increase of 2.5 mg next week and new increments of 5 mg/week to achieve glycemic targets or maximum dose (20mg/day); MF is an initial dose of 500mg to 500mg increments every 3 days to obtain the target or maximum dose of 2.5 g/day.

## Results

Thirty-six patients were randomized to the MT group and 45 for the GB group. The slurry treatment rate were 22.9% and 54.5%, respectively MP and GB group (p=0.006). Reasons for discontinuation of medications is hypoglycemia: 2.9% and 38.6% (p <0.001), respectively MF and GB group; not glycemic compensation: 2.9% and 13.6% (p=0.095), respectively MF and G groups); and gastric intolerance: 17.1% and 2.3% (p=0.021) respectively MF group and GB.

## Conclusion

Current evidence suggests that oral anti-diabetics are safe and effective for glycemic control in GD. This study was prematurely terminated by the high incidence of hypoglycemia in the GB group, considered ethically unacceptable by an independent review committee. MF was well tolerated and showed up as the first oral option in drug treatment of hyperglycemia in pregnancy.

**Figure 1:**
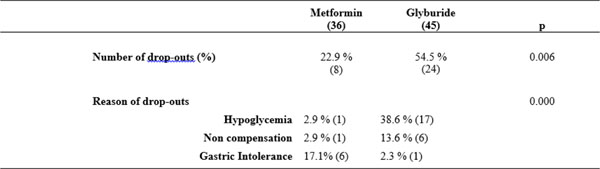
Patients' results medications.

